# Pharmacokinetics of metformin in patients with gastrointestinal intolerance

**DOI:** 10.1111/dom.13264

**Published:** 2018-03-23

**Authors:** Laura J. McCreight, Tore B. Stage, Paul Connelly, Mike Lonergan, Flemming Nielsen, Cornelia Prehn, Jerzy Adamski, Kim Brøsen, Ewan R. Pearson

**Affiliations:** ^1^ Division of Molecular and Clinical Medicine Ninewells Hospital and Medical School Dundee UK; ^2^ Department of Public Health, Clinical Pharmacology and Pharmacy University of Southern Denmark, Odense Denmark; ^3^ Institute of Experimental Genetics Genome Analysis Center Munich Germany; ^4^ German Research Centre for Environmental Health Neuherberg Germany; ^5^ Lehrstuhl für Experimentelle Genetik Technische Universität München Freising‐Weihenstephan, Munich Germany; ^6^ German Centre for Diabetes Research (DZD) Munich‐Neuherberg, Munich Germany

**Keywords:** antidiabetic drug, metformin, pharmacokinetics, type 2 diabetes

## Abstract

**Aims:**

To assess potential causes of metformin intolerance, including altered metformin uptake from the intestine, increased anaerobic glucose utilization and subsequent lactate production, altered serotonin uptake, and altered bile acid pool.

**Methods:**

For this pharmacokinetic study, we recruited 10 severely intolerant and 10 tolerant individuals, matched for age, sex and body mass index. A single 500‐mg dose of metformin was administered, with blood sampling at 12 time points over 24 hours. Blood samples were analysed for metformin, lactate, serotonin and bile acid concentrations, and compared across the phenotypes.

**Results:**

The intolerant individuals were severely intolerant to 500 mg metformin. No significant difference was identified between tolerant and intolerant cohorts in metformin pharmacokinetics: median (interquartile range [IQR]) peak concentration 2.1 (1.7‐2.3) mg/L and 2.0 (1.8‐2.2) mg/L, respectively (*P* = .76); time to peak concentration 2.5 hours; median (IQR) area under the curve (AUC)_0–24_ 16.9 (13.9‐18.6) and 13.9 (12.9‐16.8) mg/L*h, respectively (*P* = .72). Lactate concentration peaked at 3.5 hours, with mean peak concentration of 2.4 mmol/L in both cohorts (95% CI 2.0‐2.8 and 1.8‐3.0 mmol/L, respectively), and similar incremental AUC_0–24_ in each cohort: tolerant cohort 6.98 (95% CI 3.03‐10.93) and intolerant cohort 4.47 (95% CI –3.12‐12.06) mmol/L*h (*P* = .55). Neither serotonin nor bile acid concentrations were significantly different.

**Conclusions:**

Despite evidence of severe intolerance in our cohort, there was no significant difference in metformin pharmacokinetics or systemic measures of lactate, serotonin or bile acids. This suggests that metformin intolerance may be attributable to local factors within the lumen or enterocyte.

## 1 INTRODUCTION

Despite affecting up to 20% of those treated, metformin intolerance is poorly understood.^1^ Intolerance to metformin is usually characterized by gastrointestinal (GI) side effects of nausea, abdominal pain, bloating or diarrhoea. Gradual uptitration of dose after introduction of metformin or slow release preparations can, in some cases, attenuate symptoms of intolerance; however, in 5% of individuals exposed to metformin, the severity of the GI side effect leads to discontinuation of treatment.^1^ For others, metformin intolerance may result in sub‐optimal dosing or poor adherence. These factors delay optimal glycaemic control in the individual, result in the addition of, or switch to, alternative oral anti‐hyperglycaemic agents, and, as a result, potentially contribute to increased risk of microvascular complications of diabetes. Metformin is the first‐line pharmaceutical treatment for type 2 diabetes recommended by the American Diabetes Association and European Association for the Study of Diabetes guidelines.^2^ These, and other guidelines,^3^ recommend metformin based on prospective^4^, ^5^, ^6^, ^7^ and retrospective^8^ studies that demonstrate an improved glycaemic profile with metformin treatment, reduction in cardiovascular mortality,^4^, ^6^, ^7^, ^8^ no associated hypoglycaemia,^5^ and weight neutrality or weight loss.^5^ These desirable characteristics, along with their low cost, explain metformin's status as the most extensively prescribed anti‐hyperglycaemic agent worldwide. These same characteristics drive the need for ongoing research into the mechanisms underlying intolerance to metformin, aiming to prevent, modulate or treat intolerance. This would not only benefit the individual but could have significant implications for health economy.

Metformin has a complex relationship with the gastrointestinal tract.^9^ It is predominantly absorbed from the small intestine, with a bioavailability of ~60%^10^; however, it also exerts many effects on the intestine, as previously described.^9^ Multiple hypotheses for the mechanism of GI intolerance to metformin have been proposed, including abnormal uptake, increased lactate production, and accumulation of serotonin, histamine or bile acids.

Metformin uptake from the gut lumen is transporter‐dependent.^10^, ^11^ Genetic variation^12^, ^13^, ^14^, ^15^ in or inhibition^12^, ^14^ of transporters, such as organic cation transporter (OCT)1, could alter metformin uptake from the intestinal lumen to enterocytes, and subsequently affect efflux of metformin across the basolateral membrane to the systemic circulation. This would lead to changes in metformin concentration within the GI tract, enterocytes or systemic circulation.

Previous studies have shown that metformin concentration in enterocytes has been recorded at up to 300 times higher than the systemic concentration,^16^ and the variation in transporter activity described above could result in even greater differences in some individuals. Metformin is known to increase glucose uptake and anaerobic glucose utilization in the intestine, resulting in increased lactate production.^16^, ^17^, ^18^, ^19^, ^20^ In humans, there is a small but significant increase in systemic lactate when comparing those taking metformin with those who are not.^20^ We suggest that metformin intolerance may be associated with an increased concentration of metformin in the intestine, or prolonged exposure of the enterocyte to metformin, leading to a greater increase in anaerobic glucose utilization and lactate production than in tolerant individuals. The increase in local lactate concentration may contribute to the intolerance to metformin. Intracellular lactate accumulation will lead to a subsequent increase in measurable serum lactate.^20^

Metformin is known to stimulate the release of serotonin from enterochromaffin cells,^21^ and is a substrate for serotonin transporter (SERT).^14^, ^21^, ^22^ Metformin may inhibit the uptake of serotonin from the intestinal lumen, leading to accumulation of serotonin in the gut. Serotonin activates afferent neurons of the enteric nervous system, and is responsible for peristaltic and secretory reflexes within the intestine, as well as information transmission to the central nervous system.^23^ Known serotonergic effects on the gut include nausea, vomiting and diarrhoea,^24^ which are in‐keeping with the GI side effects seen in metformin intolerance. Histamine also increases gut motility,^25^ and metformin may reduce the enterocytic metabolism of histamine by diamine oxidase.^22^

It is recognized that metformin reduces ileal absorption of bile acid,^26^ leading to an increase in the bile acid pool and potential osmotic diarrhoea. Metformin could potentially alter the deconjugation of primary bile acids to secondary bile acids by bacterial 7α‐dehydroxylase^27^, ^28^, ^29^ as a result of the reduced diversity in the microbiome associated with metformin,^30^ specifically a reduction in the genera known to produce 7α‐dehydroxylase.

This open‐label pharmacokinetic study investigated these hypothesized mechanisms for metformin intolerance by studying how individuals tolerant to metformin differed from those who are intolerant. Plasma metformin and serum lactate concentrations were measured, along with targeted metabolomics, in the hours following the administration of a single dose of immediate release metformin 500 mg.

## 2 MATERIALS AND METHODS

This study was conducted in the Clinical Research Centre at Ninewells Hospital, Dundee, between June 2015 and April 2016. It was co‐sponsored by the University of Dundee and NHS Tayside, and ethical approval was given by the East of Scotland Research Ethics Committee. The study was conducted in accordance with the Good Clinical Practice guidelines, and the Declaration of Helsinki. The study was registered on the public database http://clinicaltrials.gov (identifier NCT03361878). Formal written informed consent was obtained from each individual prior to inclusion.

### 2.1 Recruitment and study design

Individuals were recruited if they had type 2 diabetes (T2D), were white European, and met the criteria for tolerance or intolerance to metformin. Metformin‐intolerant individuals were defined as those who had previously been treated with a maximum of 1000 mg metformin daily for a maximum of 8 weeks, and discontinued treatment because of GI upset (Criterion 1). Alternatively, intolerance was defined as inability to increase metformin dose above 500 mg without experiencing GI side effects, despite having a glycated haemoglobin (HbA1c) concentration >53 mmol/mol (Criterion 2). Tolerant individuals were defined as those taking 2000 mg metformin daily in divided doses, with no GI side effects. Those taking metformin were asked to discontinue their metformin 72 hours prior to the study. The length of washout period was based on an estimated t_1/2_ for plasma metformin of 5.7 hours.^10^ Exclusion criteria were: inability to consent; age not in the range of 18 to 90 years; estimated glomerular filtration rate < 60 mL/min; pregnancy; history of gastric bypass; evidence of slowed gastric or intestinal motility. None of the patients included were treated with drugs known to affect the pharmacokinetics of metformin *in vivo*,^31^ which are as follows: acarbose^32^; cephalexine^33^; cimetidine^34^; dolutegravir^35^; pyramethamine^36^; ranolazine^37^; trimethoprim^38^; and tyrosine kinase inhibitors.^39^

A total of 10 metformin‐intolerant individuals were recruited from the DIRECT cohort^40^ in Tayside, 8 of whom met intolerance Criterion 1. Ten metformin tolerant individuals were then recruited from the GoDARTS^41^ cohort, after matching for gender, age and body mass index (BMI).

Participants attended the Clinical Research Centre at Ninewells Hospital and fasted from midnight. At 9:00 am (time 0) a blood sample was obtained prior to administration of a single dose of immediate release oral metformin 500 mg. Further blood samples were taken at 0.5, 1, 1.5, 2, 2.5, 3, 3.5, 4, 6, 8 and 24 hours post‐metformin administration. Urine was collected over the 24 hours post‐metformin administration. Participants were given breakfast 2 hours and lunch 5 hours post‐metformin administration. Plasma metformin and lactate concentrations were measured at all time points, using plasma lactate concentration as a proxy of intestinal lactate production, secondary to metformin concentration within the enterocyte. Plasma lactate was measured using a lactate oxidase method; plasma and urine metformin concentrations were determined using liquid chromatography and tandem mass spectrometry (LC‐MS/MS), and the limit of quantification was 0.01 mg/L. Histamine and serotonin levels, and bile acids were determined using the targeted metabolomic assays Biocrates Absolute*IDQ* p180 Kit and Biocrates Bile Acids Kit, respectively. Full descriptions of analytical methods are provided in the Supporting Information.

During the study, a Metformin Symptom Severity Score was completed by participants (Supporting Information in File S1). This questionnaire details the individual's maximum tolerated dose of metformin, identifies which GI side effects were experienced while taking metformin, and scores the severity of the symptoms. This was completed to confirm the phenotype of the cohorts, and gather information as to the nature of the individuals' side effects. The questionnaire was not used as a diagnostic tool in the present study, but as a means of characterizing the intestinal intolerance experienced and the perceived severity of this. The “true diagnosis” of intolerance was based on the inclusion criteria alone.

### 2.2 Statistical analysis

The primary endpoint was metformin pharmacokinetics as determined by the area under the curve (AUC) of metformin concentration over time. The study was powered to detect a 30% difference in AUC_0–24_ of the metformin concentration–time curve, with 80% power, and significance of 5%. This value was chosen based on previous studies by Najib et al.,^42^ and required a cohort of 10 metformin‐intolerant individuals plus 10 metformin‐tolerant individuals. The secondary objective of the study was to determine whether systemic lactate concentration, a surrogate for metformin concentration in the enterocyte, is associated with metformin intolerance. Additional objectives included the assessment of serotonin, histamine and bile acid concentrations in acute metformin dosing.

Pharmacokinetic data were analysed using non‐compartmental analysis using the R package NCAPPC,^43^ in conjunction with the Department of Clinical Pharmacology and Pharmacy, Institute of Public Health, University of Southern Denmark. Pharmacokinetic endpoints are presented as median with interquartile range (IQR; 25th to 75th percentiles) and geometric mean ratios with 95% confidence intervals (CIs). Time to peak concentration (t_max_) was determined visually. AUC was estimated using the linear‐up logarithmic‐down method. Statistical significance was determined using the unpaired *t* test on log‐transformed data and accepted at *P* < .05. Half‐life was estimated using the terminal slope (‐k_e_) of the log‐transformed plasma metformin concentration–time curve, using the equation t_1/2_ = ln (2)/k_e_.

Renal clearance of the drug from plasma (CL_r_) was estimated using the following equation:

$${\mathrm{CL}}_{r}=\text{amount of substrate}\;{\text{in urine}}_{0–24}/\mathrm{AUC}\;{\text{of substrate}}_{0–24}$$

The apparent total clearance from plasma after oral administration (CL/F) was calculated using:

CL/F=dose/AUCof substrate

The bioavailability of metformin was not formally measured, as this requires quantification of faecal recovery of metformin, and stool samples were not obtained; however, estimated fractional drug availability (F) was calculated, by extrapolating our data to AUC_0‐inf_. By assuming that metformin is completely excreted by the kidneys, CL = CL_r_, allowing the calculation of F by:

$$F=\left({\mathrm{AUC}}_{0-\text{inf}}/{\mathrm{AUC}}_{0-24}\right)\times \left(\text{amount of metformin}\;{\text{in urine}}_{0-24}/\text{dose}\right)$$

Creatinine clearance was calculated using the Cockcroft–Gault equation using ideal body weight (IBW), and corrected for adjusted body weight (ABW = IBW + 0.4 × [actual body weight – IBW]) in those with BMI >25 kg/m^2^.

All other data were analysed using R studio, and were assessed for normality using the Shapiro Wilks method. Those data with a normal distribution are expressed as mean ± 95% CIs and were compared using unpaired *t* test with 2 tails and unequal variance. Graphic data are plotted as mean ± SEM. Those data with non‐normal distribution are expressed as medians with IQRs and compared using the non‐parametric Mann–Whitney *U* test.

Calculation of incremental AUC (iAUC) for lactate, serotonin and bile acids used the linear trapezoidal method. For the purpose of the present study and to minimize multiple testing penalties, we analysed only serotonin and histamine from the Biocrates p180 panel, and accepted values of *P* < .05 as statistically significant. For the analysis of the bile acids panel, adjusting for the Bonferroni correction, we accepted *P* < .0024.

## 3 RESULTS

### 3.1 Baseline characteristics and effect of acute dosing

All 20 participants completed the study, with no withdrawals. The baseline characteristics are listed in Table 1. The cohorts were well matched for gender, age and BMI. There was no significant difference in creatinine clearance between the cohorts. HbA1c was different in the 2 cohorts: 60.4 (53.3‐67.5) mmol/mol and 74.1 (69.0‐79.2) mmol/mol in the tolerant and intolerant cohorts, respectively, but this should not have affected the pharmacokinetics of metformin. This difference is not surprising as the intolerant cohort had discontinued metformin, and their higher HbA1c concentration may represent the difficulty in optimizing their medical management. Both cohorts had additional anti‐hyperglycaemic medications prescribed, however, including sulphonlyureas, thiazolidinediones, dipeptidyl peptidase‐4 (DPP‐4) inhibitors, glucagon‐like peptide‐1 (GLP‐1) receptor agonists and insulin. Additional medication was administered 2 hours post‐metformin dosing.

**Table 1:** Baseline characteristics

Characteristic	Metformin‐tolerant group	Metformin‐intolerant group	P
Number of participants	10	10	1.000
Female/Male	7/3	7/3	1.000
Age, years	67.5 (60.8‐72.5)	71.0 (65.75‐80.3)	.307
Age at diagnosis, years	51.5 (51.0‐58.0)	60.0 (57.3‐61.8)	.111
Diabetes duration, years	12.0 (9.0‐15.5)	12.0 (7.5‐14.8)	.850
HbA1c, mmol/mol	60.0 (55.0‐68.0)	72.0 (67.3‐76.8)	**.012**
Weight, kg	90.0 (79.0‐97.2)	91.2 (79.6‐104.0)	.910
BMI	34.6 (26.3‐38.3)	34.3 (29.5‐38.5)	.800
Creatinine clearance	86.3 (76.6‐107.3)	78.8 (68.3‐93.2)	.353
Sulphonylureas, *n*	3	6	.370
DPP‐4 inhibitors, *n*	3	1	.582
GLP‐1 receptor agonist, *n*	3	1	.582
Thiazolidinediones, *n*	0	2	.474
Insulin, *n*	4	4	1.000

Abbreviations: DPP‐4, dipeptidyl peptidase; GLP‐1 glucagon‐like peptide‐1.

Data are median (interquartile range), except where indicated otherwise. *P* value for Mann–Whitney *U* test. For categorical data, *P* value for Fisher's exact test.

The Metformin Symptom Severity Score was completed by all participants, with a potential score ranging from 0 to 50. The intolerant cohort had a mean severity score of 30.4, much greater than that of the tolerant cohort (1.9; *P* < .0001). Of the 10 tolerant individuals, 8 scored 0 for the severity score, with the 2 individuals who scored 8 and 11 having symptoms of irritable bowel syndrome which preceded metformin and were unchanged by metformin treatment. Of the intolerant cohort, 70% of participants had previously experienced nausea with metformin, 50% described abdominal pain or bloating, and 50% had diarrhoea.

**Figure 1:**
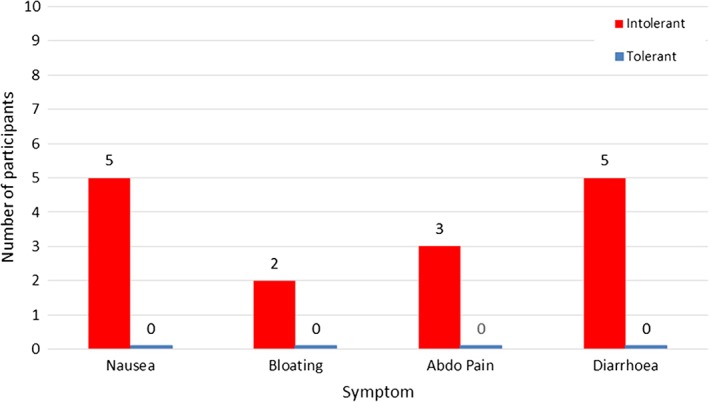
Symptoms of metformin intolerance by phenotype, after a single dose of metformin, 500 mg

During the 24‐hour study, 9 of the 10 intolerant individuals experienced GI side effects after 500 mg of metformin, while none of the tolerant cohort described any symptoms. Of the intolerant cohort, 50% had diarrhoea, 50% experienced nausea, with 30% describing abdominal pain, and 20% had bloating (Figure 1 and Table S1 in File S2). However, as this is an open‐label study, it is susceptible to reporting bias in those expecting symptoms of intolerance with metformin, with a potential over‐reporting of GI symptoms. It should also be noted that the intolerance seen in the 24‐hour study period is acute intolerance. We cannot comment on chronic intolerance, although our inclusion criteria identified individuals with true, chronic intolerance.

### 3.2 Metformin pharmacokinetics in intolerant and tolerant individuals

**Figure 2:**
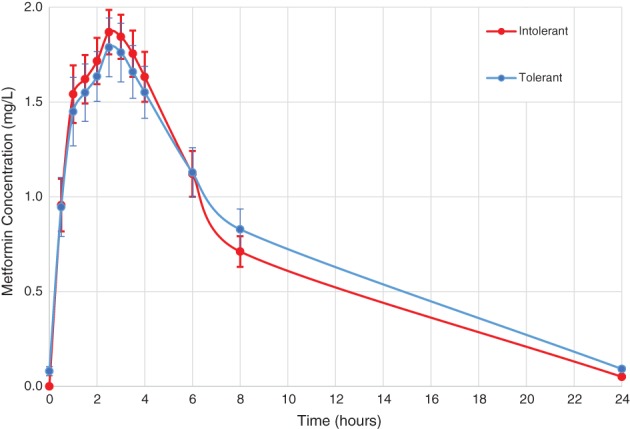
Plasma concentration of metformin over time, after a single dose of 500 mg given at time 0 hours. Data points are mean ± SEM

At time 0 hours (pre‐metformin dose) the intolerant group had a plasma metformin concentration, as expected, under the limit of detection. The metformin‐tolerant group, despite 72 hours of metformin washout, had a detectable metformin concentration, median (IQR) 0.067 (0.030‐0.095) mg/L at baseline. Similarly, at 24 hours, the median (IQR) metformin concentration in the tolerant cohort was higher (0.085 [0.066‐0.135] mg/L) than the intolerant cohort (0.051 [0.034‐0.066] mg/L). Although the differences at baseline and at 24 hours post‐metformin are significantly different from zero (*P* < .001 and *P* = .015, respectively), the levels are small when compared with the peak metformin concentration after a 500‐mg dose of metformin. Peak concentration (C_max_) for both cohorts was reached at 2.5 hours post‐dose, with a median (IQR) C_max_ of 2.1 (1.7‐2.3) mg/L and 2.0 (1.8‐2.2) mg/L for the tolerant and intolerant cohorts, respectively (*P* = .76). The plasma metformin concentrations of the groups, over 24 hours post 500 mg dose, were not significantly different, with median AUC_0–24_ 16.9 and 13.9 mg/L*h in the tolerant and intolerant cohorts, respectively (*P* = .72), as shown in Figure 2. The t_1/2_ life of metformin was higher in the tolerant group (4.8 vs 4.1 hours; *P* = .001); however, the apparent oral volume of distribution, CL/F and CL_r_ did not differ between the tolerant and intolerant groups (Table 2).

**Table 2:** Pharmacokinetic characteristics after acute metformin dosing

	Intolerant group Median (IQR)	Tolerant group Median (IQR)	Geometric mean ratio (95% CIs)	*P* value (unpaired *t* test)
AUC, mg/L*h	13.9 (12.9‐16.8)	16.9 (13.9‐18.6)	0.95 (0.72‐1.26)	.72
C_max_, mg/L	2.0 (1.8‐2.2)	2.1 (1.7‐2.3)	1.04 (0.83‐1.30)	.76
T_1/2_, h	4.1 (3.8‐4.3)	4.8 (4.7‐5.3)	0.82 (0.76‐0.89)	<.001
CL/F, L/h	35.2 (29.4‐38.1)	28.6 (25.8‐34.6)	1.07 (0.81‐1.43)	.62
V/F, L	211.4 (164.0‐225.8)	197.3 (186.0‐261.3)	0.88 (0.66‐1.17)	.36
CL_r_, L/h	17.6 (13.9‐25.5)	20.5 (14.7‐25.2)	0.88 (0.56‐1.41)	.59
F, %	71 (62‐84)	95 (56‐101)	0.83 (0.53‐1.27)	.38

Abbreviations: CL/F, apparent total clearance from plasma after oral administration; CL_r_, renal clearance of the drug from plasma; F, estimated bioavailability; V/F, volume of distribution.

### 3.3 Serum lactate and metformin intolerance

**Figure 3:**
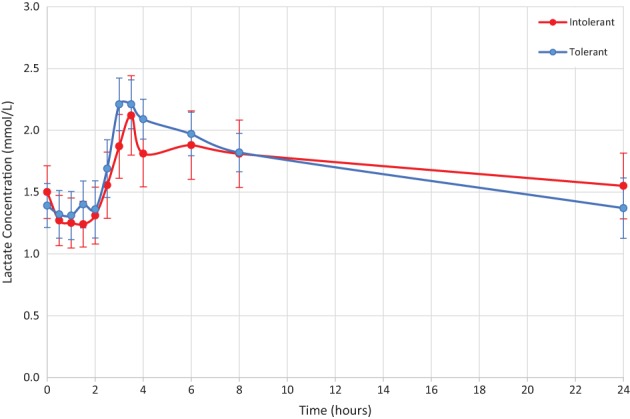
Mean lactate concentration over time, after a single dose of metformin 500 mg at time 0 hours. Data points are mean ± SEM

The lactate concentration increased post‐metformin with the median time to peak 3.5 hours post‐dose (Figure 3). Mean peak lactate concentration was 2.4 mmol/L for both groups (tolerant 95% CI 2.0‐2.8 and intolerant 95% CI 1.8‐3.0). There was no significant difference in the iAUC_0–24_ for lactate between the tolerant (6.98 mmol/L*h [95% CI 3.03‐10.93]) and intolerant (4.47 mmol/L*h [95% CI –3.12‐12.06]) groups (*P* = 0.55).

### 3.4 Plasma serotonin, histamine and bile acid concentrations

The incremental AUC_0–24_ of the serotonin concentration–time curve did not differ between the cohorts (*P* = .529), and there was no apparent rise in plasma serotonin after metformin dosing in either group (Figure S1 in File S2). Histamine levels were below the lower limit of detection in the p180 panel for both cohorts.

The Biocrates bile acid panel measures the concentration of 20 different bile acids. There was no difference in incremental AUC_0–24_ between the tolerant and intolerant cohorts for each individual bile acid, when corrected for multiple testing. Similarly, when considering the bile acids by class, primary, conjugated primary, secondary and conjugated secondary, no significant difference was identified (Table S2 in File S2).

## 4 DISCUSSION

Metformin intolerance is a common and costly challenge in the management of type 2 diabetes. Despite metformin's status as the first‐line medical treatment for type 2 diabetes, its mechanism of action is still debated. Although it is widely accepted that metformin acts in the liver to reduce gluconeogenesis,^44^ there is increasing evidence that metformin may exert some of its effect via the gastrointestinal tract,^9^ and it is unclear which of these potential mechanisms of action may be linked to metformin intolerance. In the present study of extreme intolerance, we have shown for the first time that metformin intolerance is unlikely to be mediated by differences in absorption, distribution or elimination of metformin. We also showed that intolerance was not associated with lactate derived from anaerobic glucose metabolism in the gut, altered systemic bile acid or serotonin concentration.

Metformin uptake from the intestine is predominantly via 3 transporters: OCT1; plasma membrane monoamine transporter (PMAT); and SERT. In observational studies using GoDARTS data, Dujic et al.^12^ demonstrated increased risk of metformin intolerance in those with reduced function alleles for OCT1, and latterly SERT transporters.^14^ Studies investigating the effect of OCT1 genotype on the pharmacokinetics of metformin have reported varying results. Shu et al.^45^ showed that, after acute dosing with metformin, the AUC of metformin was significantly greater in those with OCT1 variants compared with those with wild‐type OCT1. However, steady‐state pharmacokinetics of metformin appear to be independent of OCT1 genotype.^46^ Christensen et al.^15^ identified a number of SNPs in PMAT which were associated with reduced trough steady‐state metformin concentrations, significant to the *P <* .05 level, but this result did not withstand multiple testing. The above studies indicate that systemic metformin concentration may differ according to transporter genotype, and genotype has been associated with risk of intolerance; therefore, we wanted to see if systemic metformin concentration was associated with intolerance. The present study shows that, despite a well defined extreme intolerant phenotype, with 90% of the intolerant participants experiencing symptoms of metformin intolerance after a 500‐mg dose, neither the C_max_ nor t_max_ (and therefore absorption) of metformin, were significantly different between cohorts (Table 2). The lack of association of metformin pharmacokinetics with severe intolerance suggests that the association reported of OCT1 and SERT variants altering metformin intolerance may reflect an impact of these transporter variants on local rather than systemic metformin concentrations.

We identified a surprising difference in baseline metformin concentration, resulting from detectable metformin in the plasma of the tolerant group after 72 hours' washout. The detection of metformin after 72 hours washout may represent an improvement in metformin assay: from gas chromatography, to high‐performance liquid chromatography and now liquid chromatography with tandem mass spectrometry. Results from the original pharmacokinetic studies of the 1970s would suggest 72 hours without metformin should result in complete washout.^47^ The persistence of measurable plasma metformin at 72 hours is likely to be indicative of a two‐ (or more) compartment model, with metformin taken up and released slowly, for example, by erythrocytes. The slow elimination phase of metformin from the erythrocyte compartment has a t_1/2_ of 20 hours,^10^, ^47^, ^48^ compared with a plasma t_1/2_ of 5.7 hours in subjects with normal renal function.^10^ This is the likely cause of the difference in the calculated plasma t_1/2_ of the 2 cohorts, as the tolerant cohort had been at steady‐state while on metformin and probably had higher metformin accumulation in secondary compartments. By contrast, the intolerant group had depleted secondary compartments, which were absorbing some of the excess metformin and leading to a shorter elimination half‐life.

Where transporter dysfunction may lead to reduced efflux and the systemic concentration of metformin, it may also lead to increased enterocytic or intraluminal metformin concentration. Cycling of metformin between lumen and enterocyte, or uptake to enterocyte with reduced efflux, could lead to increased local metformin concentration. The resulting increase in glucose uptake and anaerobic glucose utilization, leads to a subsequent rise in intracellular lactate concentration.^16^, ^17^, ^18^, ^19^, ^20^ As intracellular lactate rises, it is released into the systemic circulation; therefore, measuring plasma lactate concentration can be used as a proxy measure of lactate production secondary to intestinal metformin concentration. Serum lactate concentration was not significantly different between tolerant and intolerant cohorts, indicating that enterocyte metformin concentration was similar in both groups. Both groups did see a rise in lactate from 2 hours, peaking at ~3.5 hours post‐dose, at a mean maximum concentration of 2.4 mmol/L, which is above the normal range in clinical practice. Portal venous sampling for lactate concentration may provide a more accurate measure of intestinal lactate production, when compared with peripheral concentrations, but this is extremely challenging to carry out in humans and beyond the scope of the present pharmacokinetic study.

The use of metabolomics to measure serotonin and bile acids gave further insight into metformin intolerance. Serotonin was detectable using the Biocrates p180 panel, but metformin dosing did not increase serotonin concentrations; however, this does not exclude a local effect of metformin on serotonin uptake by SERT. Bile acid concentrations varied post‐metformin dosing, but we did not identify a difference in systemic concentrations of the individual or grouped bile acid concentrations between tolerant and intolerant cohorts. There was a trend toward a lower total AUC for deoxycholic acid (DCA), a secondary bile acid from the conversion of cholic acid by 7α‐dehydroxylase, in the intolerant group (*P* = .052). This is interesting as most bile acids are reabsorbed in the terminal ileum, whereas DCA is absorbed from the colon.^26^ A reduced plasma concentration may indicate a reduced uptake of DCA, resulting in accumulation in the colon, which could potentially lead to bile acid diarrhoea. Further studies are required to investigate the role of the microbiome, and subsequent changes to bile acid metabolism, in metformin intolerance.

We acknowledge that the present study has a number of limitations. Firstly, the study had a small sample size, but was powered to detect a 30% change in metformin AUC between cohorts. We deemed a priori that this would be a clinically important difference when comparing such extremes of intolerance. The similarity in the mean concentrations for the 2 groups, and overlap of the distributions of individual values, are not consistent with these parameters explaining the mechanism for the marked difference in tolerance seen in these 2 groups. However, the point estimates for some of the pharmacokinetic variables and lactate do differ and this difference might achieve statistical significance if the sample size were much larger so it is possible that more subtle differences in metformin pharmacokinetics or the other measures evaluated do contribute to metformin intolerance. Secondly, we observed incomplete washout of metformin in the tolerant cohort, which highlights the need for a longer washout in future studies, but as discussed above, the metformin level at baseline was very low when compared to the peak post‐dose concentration and did not have an impact on the measures of metformin absorption. Thirdly, metformin is known to increase GLP‐1, and it is possible that this may lead to gastrointestinal symptoms in some cases; however, we were unable to measure GLP‐1 in our study cohort because of the concurrent use of DPP‐4 inhibitors and GLP‐1 receptor agonists. Finally, serum lactate concentration increased 2 hours post‐metformin dosing, but a potential confounding factor for this rise in lactate is the ingestion of a carbohydrate‐rich meal at 2 hours post‐metformin; however, previous studies in healthy volunteers indicate that the lactate concentrations increased transiently to a maximum at 90 minutes post mixed meal, returning to baseline by 180 minutes.^49^ Participants in the study received a second carbohydrate‐rich meal at 5 hours post‐metformin dosing, which did not correspond to a further peak in serum lactate level. This supports the conclusion that the rise in and peak lactate concentration is associated primarily with metformin dosing, as opposed to ingestion of a carbohydrate‐rich meal.

In conclusion, in this pharmacokinetic study of well defined extreme metformin‐intolerant and metformin‐tolerant individuals, we ruled out multiple potential systemic effects of metformin that may have contributed to metformin intolerance. We showed that the differences between tolerant and intolerant cohorts in the absorption, distribution or elimination of metformin, or in systemic lactate, serotonin or bile acid concentrations, were too small to be the mechanism of intolerance. It would be interesting to investigate further the link between transporter genotype, pharmacokinetics and tolerance of metformin, as genotype was not considered in the present study. To do so, a large recruit‐by‐genotype study would be necessary. The results from the present recruit‐by‐phenotype study suggest that metformin intolerance is likely to be mediated by local factors within the lumen or enterocyte. There is, therefore, a need to undertake more mechanistic studies that investigate the local (luminal) environment, including the microbiome, in intolerant vs tolerant individuals.

## Supporting information


**File S1.** The "Metformin Symptom Severity Score", used to characterise participants symptoms and severity of intolerance.

**File S2.** Supplementary materials
