# Supplementary material for: Pharmacokinetics of metformin in patients with gastrointestinal intolerance
Source: Diabetes Obes Metab. 2018 Mar 23;20(7):1593–601. doi: 10.1111/dom.13264 (PMC6033038; doi:10.1111/dom.13264)
Supplement: Supplementary file 1 — File S1. The "Metformin Symptom Severity Score", used to characterise participants symptoms and severity of intolerance. [file DOM-20-1593-s001.docx]

**Questionnaire to Assess Character and Severity of Metformin Intolerance**

**TREATMENT:** *SCORE*

1. Are you still taking metformin?

- Yes (if still taking skip to question 6) 0
- No 5

If you are **no longer taking** metformin:

1. Was the metformin stopped due to side-effects?

- Yes (go to question 3) 5
- No (go to question 6) 0

1. What dose were you taking at the time of discontinuing metformin?

- 500mg once daily (1 tablet once daily) 4
- 1000mg once daily (2 tablets once daily) 3
- 500mg twice daily (1 tablet twice daily) 2
- 2000mg once daily (four tablets once daily) 1
- 1000mg twice daily (two tablets twice daily) 0

1. Had you previously been on a lower dose of metformin, which you tolerated without significant side-effects?

- Yes 0
- No 1

1. If yes, what was the maximum dose you tolerated?

­­­­­­­­­­­­­­­­­­­­

­_________________________________________ 2

(if 500 mg OD)

1. Have you been trialled on a modified release preparation of metformin (e.g. Glucophage SR)?

- Yes 2
- No 0

**In the last week** **of taking metformin**, have you experienced?

1. Nausea?

- Yes 1
- No 0

1. If yes, how many times?
   - - More than once a day 4
     - Once a day 3
     - Occasionally 2
     - Only once 1
2. How would you score its severity from 1 - 5 (where 1 is mild, not causing distress or affecting your daily routine, and 5 is very severe, causing marked distress and / or disrupting your daily activities) ?

______________________________________________ __ / 5

1. Abdominal bloating / pain?

- Yes 1
- No 0

1. If yes, how often?
   - - More than once a day 4
     - Once a day 3
     - Occasionally 2
     - Only once 1
2. How would you score its severity from 1 - 5 (where 1 is mild, not causing distress or affecting your daily routine, and 5 is very severe, causing marked distress and / or disrupting your daily activities) ?

_____________________________________________ __ / 5

1. Diarrhoea?

- Yes 1
- No 0

1. If yes, how often?
   - - More than once a day 4
     - Once a day 3
     - Occasionally 2
     - Only once 1
2. How would you score its severity from 1 - 5 (where 1 is mild, not causing distress or affecting your daily routine, and 5 is very severe, causing marked distress and / or disrupting your daily activities) ?

_____________________________________________ __ / 5

1. Have you experienced any of the side-effects you have described above PRIOR to starting metformin?

- Yes -5
- No 0

1. If yes, whilst taking metformin have the symptoms:
   - - Improved -1
     - Worsened 1
     - Stayed the same 0

**TOLERANCE:**

1. Would you describe yourself as:
2. Tolerant of metformin treatment 0
3. Mildly intolerant of metformin treatment 1
4. Intolerant of metformin treatment 2
5. Severely intolerant of metformin treatment. 3

***Thank you for taking the time to complete this questionnaire.***

**For Clinical Staff Use:**

TOTAL SCORE: ____ / 50

Score:

0 – 10 = tolerant (T)

11-20 = mild intolerance (MI)

21-30 = intolerant (I)

31-50 = severely intolerant (SI)

| Severity Score  ( __/50 ) | Score based tolerance  ( T / MI / I / SI ) | Patient perceived tolerance  ( T / MI / I / SI ) | Correlation  ( Y / N ) |
| --- | --- | --- | --- |
|  |  |  |  |
