# Supplementary material for: Pharmacokinetics of metformin in patients with gastrointestinal intolerance
Source: Diabetes Obes Metab. 2018 Mar 23;20(7):1593–601. doi: 10.1111/dom.13264 (PMC6033038; doi:10.1111/dom.13264)
Supplement: Supplementary file 2 — File S2. Supplementary materials [file DOM-20-1593-s002.docx]

# Supplementary methods

### Determination of metformin concentrations in plasma and urine samples.

The concentration of metformin in plasma and urine samples were determined at the Department of Clinical Pharmacology and Pharmacy, Institute of Public Health, University of Southern Denmark, by use of liquid chromatography and tandem mass spectrometry (LC-MS/MS). The LC-MS/MS system consisted of an Ultimate 3000 UHPLC system connected to a TSQ Quantiva Triple Quadropole Mass Spectrometer with heated electrospray ionization (Thermo Scientific, San Jose, CA). Data acquisition was performed in single reaction monitoring (SRM) mode. Metformin was quantitated by positive ionisation at the transition from (m/z) 130.4 – 71.1, and with (m/z) 130.4 – 60.1 as a qualifier trace. Metformin-d6 (internal standard) was monitored from (m/z) 136.4 – 77.1. The analytical separation was performed using hydrophilic interaction chromatography as described by Nielsen et al.^1^

The sample preparation of the plasma samples consisted of a single protein precipitation step. To a 100μL plasma sample, 10 μL 25 ug/mL metformin-d6 (internal standard), 20 μL 0.53M ammonium acetate and 390 μL acetonitrile were added. The sample was vortex mixed for 30 sec and centrifuged at 3.000g for 15 minutes. The urine samples were diluted (1:50) before use, but were otherwise treated as the plasma samples. A volume of 10 μL of the supernatant was injected onto the LC-MS/MS system. Calibration curves, as well as quality control samples, were prepared and included in each batch of analysis. The intra- and interday variability was < 8%. The limit of detection (LOD) for the method was 1 ng/mL and limit of quantification (LOQ) was 10 ng/mL.

### Determination of histamine, serotonin and bile acid concentrations

EDTA plasma samples from time points 0, 3.5, 8 and 24 hours post-metformin were used for targeted metabolomics measurements, which were done in the Metabolomics Platform of the Genome Analysis Center, Helmholtz Zentrum München). Histamine and serotonin were quantified using the Biocrates Absolute*IDQ*^TM^ p180 Kit and bile acids using the Biocrates^TM^ Bile Acids Kit (BIOCRATES Life Sciences AG, Innsbruck, Austria).

The measurements of plasma samples with the Absolute*IDQ*^TM^ p180 Kit and FIA- and LC-ESI-MS/MS (flow injection analysis-/liquid chromatography-electrospray ionisation-tandem mass spectrometry) have been described in full detail by Zukunft et al.^2^ Out of 10 µL sample, 188 metabolites can be quantified simultaneously. The assay includes free carnitine, 39 acylcarnitines, 21 amino acids, 21 biogenic amines, hexoses, 90 glycerophospholipids, and 15 sphingolipids.

Using the Biocrates^TM^ Bile Acids Kit and LC-ESI-MS/MS, 20 bile acids can be quantified out of 10 µL plasma. The assay includes cholic acid, chenodeoxycholic acid, deoxycholic acid, glycocholic acid, glycochenodeoxycholic acid, glycodeoxycholic acid, glycolithocholic acid, glycoursodeoxycholic acid, hyodeoxycholic acid, lithocholic acid, alpha-muricholic acid, beta-muricholic acid, omega-murichoclic acid, taurocholic acid, taurochenodeoxycholic acid, taurodeoxycholic acid, taurolithocholic acid, tauromuricholic acid (sum of alpha and beta), tauroursodeoxycholic acid, ursodeoxycholic acid. Compound identification and quantification were based on scheduled multiple reaction monitoring measurements (sMRM). We provide the description on how sample preparation and mass spectrometric measurements were performed in the laboratory.^3^ In short, 10 µL of internal standard solution in methanol were pipetted onto the filter inserts of a 96 well sandwich plate. After drying the filters for 5 min at RT in a nitrogen stream, 10 µL of blank, calibration standards, quality control samples and plasma samples were pipetted into the distinct respective wells and the filters were dried again for 5 min. For extraction of metabolites and internal standards, 100 µL of methanol were added and the plate was shaken for 20 min at 650 rpm. The metabolite extracts were eluted to the lower deep well plate by a centrifugation step (5 min at 500 x *g* at RT). The upper filter plate was removed, the extracts were diluted with 60 µL ultrapure water, and the plate was shaken for 5 min at 450 rpm and placed into the cooled auto sampler (10 °C) for LC-MS/MS measurements. The LC-separation was performed using 10 mM ammonium acetate in a mixture of ultrapure water/formic acid v/v 99.85/0.15 as mobile phase A and 10 mM ammonium acetate in a mixture of methanol/acetonitrile/ultrapure water/formic acid v/v/v/v 30/65/4.85/0.15 as mobile phase B. Bile acids were separated on the UHPLC column for Biocrates^TM^ Bile Acids Kit (Product No. 91220052120868) combined with the precolumn SecurityGuard ULTRA Cartridge C18/XB-C18 (for 2.1 mm ID column, Phenomenex Cat. No. AJ0-8782). All solvents that have been used for sample preparation and measurement were of HPLC grade.

Both Biocrates methods has been proven to be in conformance with the EMEA-Guideline "Guideline on bioanalytical method validation” (July 21st 2011)^53^ which implies proof of reproducibility within a given error range. Sample preparation and FIA- and LC-MS/MS measurements were performed as described by the manufacturer in the manuals UM-P180 and UM-BA. Analytical specifications for LOD (limit of detection), LLOQ and ULOQ (lower and upper limit of quantification), specificity, linearity, precision, accuracy, reproducibility, and stability were described in Biocrates manuals AS-P180 and AS-BA. The LODs were set to three times the values of the zero samples (PBS). The LLOQ and ULOQ were determined experimentally by Biocrates. Samples were handled using a Hamilton Microlab STAR^TM^ robot (Hamilton Bonaduz AG, Bonaduz, Switzerland) and a Ultravap nitrogen evaporator (Porvair Sciences, Leatherhead, U.K.), beside standard laboratory equipment. Mass spectrometric analyses were done on an API 4000 triple quadrupole system (Sciex Deutschland GmbH, Darmstadt, Germany) equipped with a 1200 Series HPLC (Agilent Technologies Deutschland GmbH, Böblingen, Germany) and a HTC PAL auto sampler (CTC Analytics, Zwingen, Switzerland) controlled by the software Analyst 1.6.2. Data evaluation for quantification of metabolite concentrations and quality assessment was performed with the software MultiQuant 3.0.1 (Sciex) and the Met*IDQ*™ software package, which is an integral part of the Absolute*IDQ*™ and Biocrates^TM^ Bile Acids Kits. Metabolite concentrations were calculated using internal standards and reported in µM.

### Determination of plasma lactate concentrations

Fluoride oxalate samples were analysed for plasma lactate using a lactate oxidase method on a Siemens ADVIA 2400 Chemistry System (analysed by Biochemical Medicine, Department of Blood Sciences, NHS Tayside).

# Supplementary Tables

*Supplementary Table 1: Metformin associated gastrointestinal side-effects after acute dosing*

| Study ID | Symptom | | | |
| --- | --- | --- | --- | --- |
|  | Nausea | Bloating | Abdominal Pain | Diarrhoea |
| MI 01 | N | N | Y | Y |
| MI 02 | N | N | N | Y |
| MI 03 | Y | N | N | N |
| MI 04 | Y | Y | N | Y |
| MI 05 | N | N | N | Y |
| MI 06 | N | N | N | N |
| MI 07 | N | Y | Y | N |
| MI 08 | Y | N | Y | Y |
| MI 09 | Y | N | N | N |
| MI 10 | Y | N | N | N |
|  |  |  |  |  |
| MT 01 | N | N | N | N |
| MT 02 | N | N | N | N |
| MT 03 | N | N | N | N |
| MT 04 | N | N | N | N |
| MT 05 | N | N | N | N |
| MT 06 | N | N | N | N |
| MT 07 | N | N | N | N |
| MT 08 | N | N | N | N |
| MT 09 | N | N | N | N |
| MT 10 | N | N | N | N |

MI = Metformin Intolerant, MT = Metformin Tolerant.

*Supplementary Table 2: Comparison of incremental AUC of bile acids after acute metformin dosing, between tolerant and intolerant cohorts. Data shown as median (IQR 25^th^ – 75^th^), µM*hr. Bonferroni correction, significance = P<0.0024. Three of the twenty measured bile acids were below the LOD, and therefore not analysed.*

| Bile acid (abbreviation) | MI | MT | Mann Whitney test |
| --- | --- | --- | --- |
| Cholic acid  (CA) | -0.73  (-3.3 – 0.71) | 0.46  (-0.04 – 2.68) | 0.1431 |
| Chenodeoxycholic acid (CDCA) | -0.06  (-3.36 – 5.78 | 0.28  (-5.02 – 4.68) | 0.8534 |
| Deoxycholic acid  (DCA) | 1.12  (-3.11 – 4.12) | 2.23  (-5.13 – 8.04) | 0.5787 |
| Glycocholic acid  (GCA) | 4.18  (1.38 – 9.99) | 1.12  (-0.92 – 4.83) | 0.0892 |
| Glycochenodeoxycholic acid  (GCDCA) | 15.84  (5.63 – 27.04) | 9.28  (-5.80 – 25.81) | 0.3527 |
| Glycodeoxycholic acid  (GDCA) | 7.65  (5.55 – 12.53) | 3.75  (-9.87 – 15.11) | 0.2799 |
| Glycolithocholic acid  (GLCA) | 0.44  (0.09 – 0.57) | 0.15  (-0.39 – 1.21) | 0.4813 |
| Glycoursodeoxycholic acid (GUDCA) | 0.66  (0.18 – 3.53) | 0.14  (-0.86 – 1.99) | 0.2799 |
| Lithocholic acid  (LCA) | -0.07  (-0.34 – 0.18) | 0.01  (-0.30 – 0.20) | 0.7054 |
| Muricholic acid, beta (MCA(b)) | 0.07  (-0.14 – 0.19) | -0.01  (-0.15 – 0.10) | 0.4813 |
| Taurocholic acid  (TCA) | 0.93  (0.23 – 2.13) | -0.01  (-0.19 – 0.61) | 0.01854 |
| Taurochenodeoxycholic acid (TCDCA) | 2.21  (0.64 – 2.78) | 0.46  (-0.45 – 1.61) | 0.04326 |
| Taurodeoxycholic acid  (TDCA) | 0.86  (0.63 – 1.52) | 0.04  (-0.52 – 1.25) | 0.07526 |
| Taurolithocholic acid  (TLCA) | 0.07  (0 – 0.20) | 0.02  (0 – 0.18) | 0.5148 |
| Tauromuricholic acid (a + b) (TMCA(a+b)) | 0.11  (0.06 – 0.18) | 0.00  (-0.11 – 0.06) | 0.01469 |
| Tauroursodeoxycholic acid (TUDCA) | 0.07  (0.03 – 0.19) | 0.02  (-0.04 – 0.09) | 0.1211 |
| Ursodeoxycholic acid  (UDCA) | 0.17  (-0.84 – 0.53) | 0.10  (-0.11 – 1.16) | 0.9118 |
| Total Primary | -2.05  (-3.83 – 0.75) | 0.57  (-4.88 – 6.91) | 0.2176 |
| Total Conjugated Primary | 0.89  (-3.59 – 4.53) | 3.80  (-5.35 – 8.72) | 0.2176 |
| Total Secondary | 24.19  (10.19 – 43.22) | 11.00  (-7.20 – 31.46) | 0.4813 |
| Total Conjugated Secondary | 11.02  (7.63 – 17.93) | 5.19  (-11.09 – 19.50) | 0.315 |

# Supplementary Figures

*Supplementary Figure 1: Mean serotonin concentration over time, following a single dose of metformin, 500mg at time 0hr. Data points are mean ±SEM.*


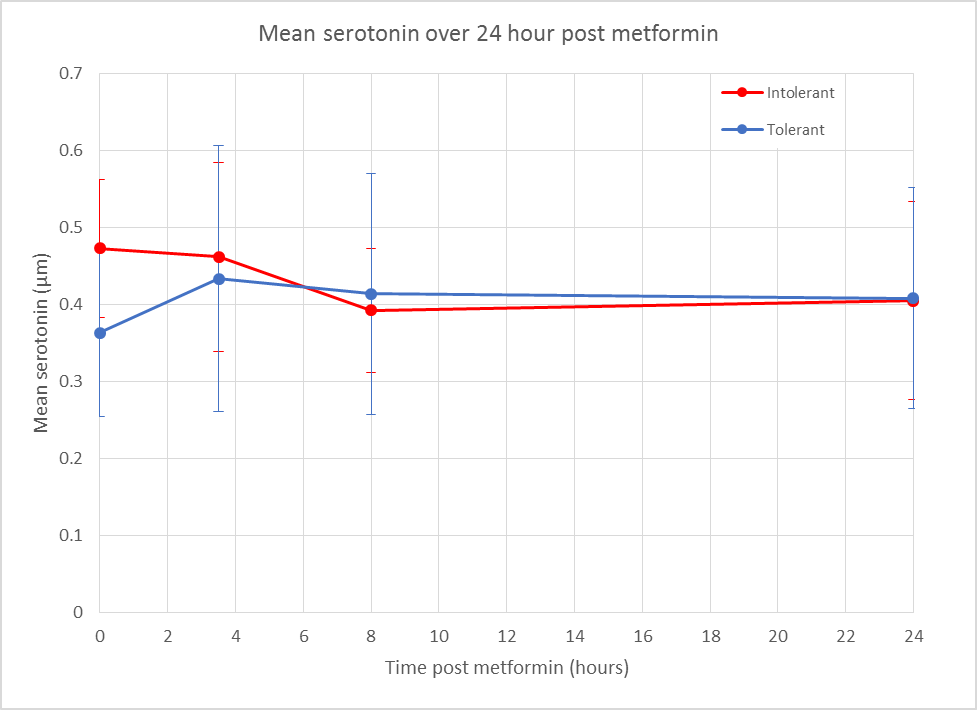


**References**

1. Nielsen F, Christensen MMH, Brøsen K. Quantitation of metformin in human plasma and urine by hydrophilic interaction liquid chromatography and application to a pharmacokinetic study. Ther Drug Monit. 2014 Apr;36(2):211-217. <https://doi.org/10.1097/FTD.0b013e3182a4598a>.
2. Zukunft S, Sorgenfrei M, Prehn C, Möller G, Adamski J. Targeted metabolomics of dried blood spot extracts. Chromatographia. 2013; 76:1295-1305. <https://doi.org/10.1007/s10337-013-2429-3>.
3. Pham HT, Arnhard K, Asad YJ, et al. Inter-laboratory robustness of next-generation bile acid study in mice and humans: international ring trial involving 12 laboratories. J Appl Lab Med. 2016;129-142.
4. Committee for Medicinal Products for Human Use (CHMP). Guideline Q19 on bioanalytical method validation. EMEA/CHMP/EWP/192217/2009 Rev 1 Corr 2, July 21, 2011. <http://www.ema.europa.eu/docs/en_GB/document_library/Scientific_guideline/2011/08/WC500109686.pdf>.
